# A guide to evaluating systematic reviews for the busy clinicians or reluctant readers

**DOI:** 10.1186/s12998-023-00501-4

**Published:** 2023-09-20

**Authors:** Stanley Innes, Charlotte Leboeuf-Yde

**Affiliations:** 1 Adult Mental Health and Wellbeing, Eastern Health, Monash University, Melbourne, Australia; 2 Department of Regional Health Research, University of Southern Denmark, Winsløwparken 19, 5000 Odense, Denmark

**Keywords:** Systematic review, Evaluation studies, Evidence-based medicine, Health personnel, Information literacy

## Abstract

Systematic reviews (SRs) provide a solution to handle information overload for busy clinicians by summarising and synthesizing studies on a specific issue. However, because SRs are complicated and often boring to read, the busy or reluctant reader may make do with the abstract. When, as it has been shown, many authors overstate efficacy or understate harm in their abstracts, not consulting the underlying article could be misleading. This means that the prudent reader must have the ability to identify the ‘tender points’ of SRs to avoid falling for ‘spin’. To this end we briefly review the method of SRs and ways to relatively quickly determine trustworthiness.

## Background

There has been an almost exponential growth in medical science-related journal publications [1], but scientific reports, in general, are not reader-friendly. For many clinicians the systematic review (SR) is, therefore, very helpful, as it summarizes the available evidence from the relevant literature.

Further, SRs are considered the most valuable type of research, placed at the top of the evidence pyramid [2], making them trusted and used to create guidelines and other policy documents. For the clinician, they represent a gold mine of information, all collected and reported in one place.

Unfortunately, though, anybody reading SRs will soon realise that all are not equally well performed. Therefore, results might have to be interpreted with caution [3]. This premise applies for all areas of health, including musculoskeletal conditions [4]. Also, consideration should be given to the possibility that an SR could be well performed but not well reported. Another problem is that SRs are often compact, technical, and quite boring to read, for which reason it is tempting for busy clinicians to read only the abstract, and they often do [5].

Unfortunately, abstracts of SRs have repeatedly been found to be deceptive, in that they may misrepresent their results by either exaggerating the good or diminishing the bad outcomes. This is called “spin” and has been shown to be particularly common for studies on low back pain with approximately 80% of abstracts being guilty of ‘spin’ [6]. Admittedly, this may not be life-threatening, but a study of SRs in emergency medicine showed a ‘spin’ percentage of approximately 30% [7], which is much more serious for patients.

This implies that the reader must be able to discern the trustable from the not so trustable SRs and be aware of the danger of reading the abstract only. To this end we will outline the key characteristics of a good quality SR and point out the ‘tender’ points. This will provide a relatively short and easy manual on SRs for the non-researcher, making it possible to relatively quickly assess, from a technical viewpoint, whether a SR is worth reading (and trusting) or not.

## Key characteristics of a good quality systematic review

### The major types of reviews

Reviews can be divided into three main groups: narrative, systematic, and other transparent types.

- **Narrative reviews** simply tell a story and may or may not have some transparent elements, but most commonly they consist of a summary of a topic, which can be both relevant and interesting. In fact, a topic may not be possible to deal with satisfactorily unless in a narrative form, as shown in this example which aimed to describe the outcomes, barriers, and facilitators relating to interprofessional practice involving chiropractors [8]. Unless the review is completely transparent, though, the reader cannot tell if what is written is also ‘true’ (including all relevant evidence, objective, and well-balanced).
- **Systematic reviews**, however, are inclusive and transparent. They can be purely descriptive or analytical and be with or without a critical element. However, increasingly the term ‘systematic’ seems to indicate that the review has also a critical view on the articles that were scrutinized. It is common that they deal with only one type of study design, but mixed design reviews are also seen, as in this example which reviews the impact of outcome measurements in clinical practice [9].
- **Many other types of transparent reviews.** There are many other types of semi-systematic or, at least, transparent reviews. These differ on how the literature is searched, the presence of quality appraisal, the approach to the analysis/synthesis, along with their presentation of results [10]. An example is the scoping review, which is increasingly often seen. It has a less rigorous approach to the search, may deal with several research designs, and lacks a critical element, because its purpose is to provide an overview over a topic without a stringent analysis, often because the topic is poorly understood and or confusing. A scoping review on Chiropractic Functional Neurology [11] is an example of how to approach a poorly defined clinical method that has only few scientific publications.

### Systematic reviews are stringent research projects

A SR has many elements in common with an ‘ordinary’ research project, such as clear pre-hoc research questions, systematic collection of data, transparent analysis of data, and objective interpretation of the results [12]. Further, the data must be trustworthy, and this depends on two things: the quality of the data, i.e., the skills of the researchers of the original studies, and the quality of the review process, i.e., the skills of the reviewers. Many journals refuse to publish SRs if a detailed design or plan (protocol) has not been registered in a relevant research register, such as PROSPERO or INPLASY. Before registration, personnel at these registers will check the protocols, which constitutes a form of quality assurance.

### The three steps of a systematic review

The method of data collection and analysis of SRs consist of three distinct steps:

i) Finding the articles.
ii) Extracting the data.
iii) Analyzing/synthesizing the data.

#### Finding the articles

It is important to find and include *all* relevant literature [13]. This is best done by searching in the right places, using the right search terms, and aiming for a relevant time period. It may also be necessary to limit the search to certain foreign languages, depending on the language skills of the reviewers and assuming they do not trust Google translate. The research report must explain all this in such detail that somebody else can do the same search and find the same articles. Although anybody can look for articles on the Internet, to do it properly requires expertise to choose relevant databases and search terms. Therefore, the assistance of a research librarian is very helpful, perhaps even necessary.

The original search will result in a list of titles and abstracts of possibly suitable articles. Usually, this list is large, perhaps containing many thousand articles. The reviewers must therefore spend quite some time, meticulously selecting the relevant ones, according to pre-determined *inclusion criteria*, such as the relevant disease, the type of treatment and control treatment, depending on the topic and type of study design. *Exclusion criteria* are also defined in advance, such as not wanting to include animal studies. No articles are to be excluded at this stage because they are of poor quality, and certainly not because they provide the ‘wrong’ or ‘unexpected’ answers / outcomes. These extracted articles will correspond to the study subjects in a clinical study, and they will provide the data used to answer the research questions.

#### Extracting the data

Three types of data are usually extracted from these articles, listed in tables in an abbreviated form, to make the analysis perfectly *transparent.* These are: (i) descriptive information, which gives an overview of the reviewed articles on factors that may influence our understanding of the outcomes, (ii) information on various items that can be suspected of inducing bias or other quality information that may influence the credibility of data, and (iii) the variables (results) used to find answers to the research question(s).

All ‘evidence’ should be included in the report, i.e., the tables that (i) describe the articles, (ii) summarize the risk of bias for each article, and (iii) the raw data that provide the background for the results. Some articles scrutinize the technical quality instead of or in addition to the risk of bias. Sometimes these tables are presented in additional files or must be downloaded from the Internet.

#### Analyzing /synthesizing of data

The collected information can be analyzed (synthesized is another term) in several ways. The best-known analytic method is probably the meta-analysis, in which estimates from the articles are combined and re-analyzed with a specific statistical method to obtain a new summary estimate surrounded by a range of values that describes the uncertainty surrounding it (confidence interval). This confidence interval will typically be much smaller than the ones in the individual studies, as there will now be many more participants in the ‘meta-study’. The meta-analysis is often used to compare outcomes in two types of treatments, when there are several studies that approached the topic in similar ways. It can also be used to establish the prevalence of something in studies that define the study population and the outcome variable similarly.

Other analytical methods could be to count the number of studies that obtained one specific response vs. those finding something else. Such as, “eight of the ten included studies found no association between the habitual use of chewing gum and jaw pain”.

Results can also be presented as numbers, percentages, odds ratios, and suchlike to be summarized into a big picture.

Other analytical approaches could be to identify common concepts or approaches and to report this narratively. A narrative report of the results is not to be confused with a narrative review, which was explained under “Three major types of reviews”.

### The critical aspect: checking if the extracted articles can be trusted

There is a strict procedure for how SRs should be performed. Part of this procedure deals with the trustworthiness of the results. This is pivotal and must be part of the analytical approach [14]. The credibility of the findings in an article depends on whether they are likely to be correctly obtained during the (original) research procedure (internal validity) and whether they are likely to be typical of the ‘real world’ (external validity). The risk of bias and other quality aspects of the methods used and the results reported are therefore scrutinized in properly conducted SRs.

#### Risk of bias

The skills of the original researchers in the extracted studies are thus assessed in a systematic manner, checking for points of (possible) bias that can arise e.g., when selecting study participants, treating these (if this is done), and summarizing the findings in the statistical analysis. ‘Bias’ is defined as an error that tends to push or distort results in a specific direction, i.e., resulting in a ‘systematic error’. Since it is not always possible to see if such an error occurs, one looks for the *risk* of it happening, i.e., “risk of bias”, referred to as ‘RoB’ in research articles. This is typically reported in tables (checklists, grids) with or without colors (green for low, yellow for moderate, and red for high RoB).

Different study designs require different research methods, which can result in different possibilities for bias. Therefore, there exist many different checklists for RoB assessment, each relevant for the various research designs (e.g., clinical trials, outcome, qualitative and animal experimentation studies) [15]. When no good checklist exists, or if there is one that needs some adaptations to meet the purposes of the study, the reviewer must design or amend an existing one in a transparent manner [16].

#### Quality issues

Errors that occur in a study are not necessarily ‘systematic’ (i.e., risking the influence of bias) but can also result in haphazard findings. One example is an inexperienced nurse responsible for taking the blood pressure, which results in nonsense (in any direction) blood pressure values. Another example could be a faulty questionnaire that lacks an appropriate answering category, with responders reacting in different, erratic ways to make it possible to provide an answer anyway. Checklists relating to quality items would probably have to be ‘invented’ by the research team, as they do not exist as prototypes. The quality of the study will *also* influence its credibility and its results, but this aspect is less often dealt with in SRs. Most authors concentrate on RoB.

#### Drawing conclusions on the RoB /quality

It is important, though, that these checklists (on RoB and possibly on quality) are carefully filled out and presented in the article for the readers to be able to see themselves if the included articles can be trusted or not. Thus, *each article* is defined as having a low, moderate, or high RoB, with the latter obviously being less trustworthy than the first. When the quality, rather than RoB, is under scrutiny, as would be the case in very technical studies, such as in laboratory studies, the same approach should be taken, defining studies as having low, moderate, or high quality. The cut-points, for when an item is ‘good’ or ‘bad’ must be pre-defined and explained in the Methods section. Their respective importance may differ with different study designs.

The *whole reviewed research area* can also be summarized in the same way, indicating if the evidence, in general, is trustable or not, using the concepts of RoB and/or quality. This is important, as poorly conducted research is more prone to bias and is therefore more likely to distort the true outcome of a study often resulting in ‘good’ outcomes, whereas well-conducted studies often have ‘poor’ outcomes, as was so dramatically demonstrated in an SR on spinal manipulation in the treatment of non-musculoskeletal disorders, where all the studies of low RoB revealed there to be no ‘effect’ of the treatment, whereas all the others reported good outcomes [17].

### Taking into account the risk of bias and/or quality in the analysis

Whatever the method used to analyze/synthesize the data, it is important not to trust all results equally. The studies with a high RoB and/or of poor quality should not be considered equal to those done properly [18]. This means that one should discern whether or not the authors have either (i) separated the ‘good’ from the ‘bad’ articles and also reported the findings from these separately whilst indicating if the results are believable or not, (ii) or summarized/calculated the results whilst including *all* the articles – good and bad - and then labelled the research *area* (i.e., all the articles) as trustable, less trustable, or not trustable at all (the GRADE method). An example of the first approach is the SR on spinal manipulation and non-musculoskeletal conditions [17], whereas a SR on the potential effect of spinal manipulation on the autonomic nervous system used the GRADE approach [19].

Incredibly, many reviewers go through the tedious work of checking their collected articles for RoB (or possibly quality) and may even report the findings in a table to then forget all about it when drawing their conclusions on the results! This is particularly common in meta-analyses, where instead of checking what happens with the final estimate when the ‘bad’ studies have been removed or discussing the credibility of the whole research area, all studies are often pooled into one happy family, thus not taking into account at all the issue of trustworthiness. One could say that this omission is what separates a ‘research technician’ from a researcher, as ‘real’ researchers, as well as enlightened readers, are willing to accept and trust studies using ‘good’ methods and not only looking for the studies with the ‘good’ results. The inclusion of less robust/trustable study results risks leading to unreliable, often inflated estimates and can result in inaccurate recommendations influencing clinical practice.

## Assessing the technical quality of a systematic review- checking for weaknesses at the ‘tender points’

A SR should have the same approach as in ‘ordinary’ research reports when interpreting results in the light of any methodological issues that could have influenced these. However, authors of SRs must deal with *two* layers of methodological issues: that of the others (i.e., the authors of the reviewed articles) and that of their own work. RoB and quality assessments take care of the first aspect, but it is important for the authors also to scrutinize the quality of their own review. Obviously, it is easier to find faults with others than with oneself, for which reason authors often seem to forget to take a critical look at their own activities. Therefore, it is often up to the reader to be critical because also SRs can have RoB and quality issues. Before reading the whole article (or perhaps ‘cheating’ by reading only the conclusion), it is, therefore, important to know if the SR was done ‘properly’, and some of this can be discerned by looking for some specific ‘tender points’. As a minimum, these should be acceptable. They are easy to detect also for the non-expert, and all readers should look for these.

Technical issues, which can result in both bias and quality issues in the review process itself, typically occur in four places, namely: (i) during the search for relevant articles, (ii) when extracting data, (iii) when analyzing the data, and (iv) in the interpretation of the results, such as producing a conclusion that can be described as “spin”.

I. Searching for relevant articles: Thus, when searching for articles, a librarian does this best. The screening of articles should be performed by two persons, independently of each other, who then adjust any divergent findings afterwards in discussions. A third person, a referee, might be needed if the two reviewers cannot obtain consensus. If one person does this, as is often the case, this should either be done twice or with somebody checking the findings afterwards. The search terms should be listed somewhere in the text (or in an additional file) and the number of articles at each stage of the screening process should be presented in a flow-chart.
II. Extracting data: Also this should be done by two people, independently of each other, as research texts can be complicated and may be misunderstood. Further, all extracted data should be visible in the results tables to make it possible for readers to check the information, theoretically making it possible to ensure there are no errors that could influence the results of the analyses. If the reader is unable to re-analyze the data based on the information in the various tables, the article is not fully transparent, and full transparency is the hallmark of a SR.
III. Analysis of data: A major problem, seen quite often in SRs, is the disrespect for the issues of bias and/or quality during the *analysis/synthesis of data*. This is noticeable in three major ways: (i) there is no RoB assessment, although there quite clearly should be one, (ii) there could be a mention of a RoB assessment but no table that shows the results, neither in the main text nor in an additional file, or (iii) there is a RoB table but no explanation of how (if at all!) this had any consequences on the data synthesis and/or interpretation. It is difficult to understand why the reviewers went to all the trouble of extracting data on RoB/quality to then forget about it. The danger here is that readers might be so confused by the multitude of details that they lose the big picture, not realizing that RoB/quality played no role in the analysis or interpretation of data.
IV. Interpretation of the results and risk of ‘spin’: Finally, the summary of findings at the beginning of the Discussion and in the Conclusions should correctly reflect the findings, which – in turn - should ultimately relate to the research questions posed at the end of the Background section. If these do not align, you know that you are dealing with an unskilled reviewer, and you have reasons to be cautious, as this mismatch may result in ‘spin’. A look at the affiliations of the authors of the review, including a search for any reported or unreported conflicts of interest, can also be helpful when watching for ‘spin’, as various types of obvious or less obvious conflicts of interest can influence biomedical research in important ways [20], also in SRs.

### Additional help to the non-expert reader

As mentioned, there are checklists for most research designs both on how to conduct a study (mainly RoB tools) but also on how to write a report (writing guidelines are found on the Equator network). The same is true for SRs. Thus, for those who wish to penetrate deeper into this topic, there is a RoB checklist for SRs, called AMSTAR [21] and a writing guideline, the PRISMA guidelines [22]. It is usually a sign of reassurance when the SR contains a reference to this guideline.

Nevertheless, as previously mentioned, the technical quality of SRs is quite often not up to standard, which could mean that the results are inaccurate. For this reason, it is important for the reader to be able to quickly scrutinize such texts before investing reading time. For this reason, we include a list of ‘tender points’ to look for in SRs (Table 1). These can be detected by searching for these items in the text without spending time on actually reading it from the beginning to the end. Most of the relevant information to look for is found in the Methods section of the SR.

**Table 1:** Some common crucial points that readers should check to see if the systematic review is credible

Main topic	Was this clearly described?	Aspects to watch out for
*Purpose of review*	Clear research questions	Are they possible to answer and not too vague?
*Finding the literature*	A clear description of the search criteria The screening processes	Were the search criteria so clearly explained that the same articles can be found by somebody else? Was the search assisted by an expert librarian? Did at least two people search and screen, blind to each other’s findings using clear inclusion/exclusion criteria?
*Obtaining information from articles*	Extraction of three types of information: I. descriptive, II. risk of bias/level of quality, III. results The tool to extract risk of bias/quality information	Was the extraction of data done by more than one person, and blindly, and is information transparently reported in tables?
*Analysis/synthesis of data*	An explanation of how the data would be analyzed (in the Methods section)	Was the analysis so clearly explained that somebody else would be able to get the same results, using the data presented in the tables? Was it shown how information on RoB/quality was used in terms of what was acceptable and how the level of acceptability influenced the analysis of data or the interpretation of the results?
*Results*	A full report of the result, no more no less.	Are the research questions answered and if this was not possible, was it explained why not?
*“Summary of findings and conclusions” in the Discussion and Conclusions in the Abstract*	The Summary of Findings (at the beginning of the Discussion) and the Conclusions (at the end of the Discussion or in the Abstract) all agree and they report also ‘uncomfortable’ results.	Are there any signs of positive or negative ‘spin’ in the Conclusion of the main text or in the Abstract?
		Do you trust these authors? If so, now read the Results. If not, ignore the results, even if they correspond to what you prefer to see!

A final consideration is one of the importance of the results [23]. That is, can the results be used in clinical practice?

## Conclusion

It is important to note that even an impressive-looking systematic review can have limitations and that it’s important to resist the temptation of reading only the Conclusions. Instead, we recommend that readers follow our brief checklist, looking for the “tender points” that will indicate whether the results can be trusted or not. These tender points are particularly noticeable in the (i) article selection, (ii) data extraction, (iii) data analysis (which all may impact the trustworthiness of the reviewed literature), and in the (iv) authors’ interpretation of the results, which should not show any signs of ‘spin’.

## Data Availability

Not applicable.

## List of abbreviations

RoB: Risk of Bias
SR: Systematic review
